# SARS-CoV-2 Infection in Beaver Farm, Mongolia, 2021

**DOI:** 10.3201/eid3002.231318

**Published:** 2024-02

**Authors:** Taichiro Takemura, Ulaankhuu Ankhanbaatar, Tirumala Bharani K. Settypalli, Dulam Purevtseren, Gansukh Shura, Batchuluun Damdinjav, Hatem Ouled Ahmed Ben Ali, William G Dundon, Giovanni Cattoli, Charles E. Lamien

**Affiliations:** International Atomic Energy Agency, Seibersdorf, Austria (T. Takemura, T.B.K. Settypalli, H.O.A.B. Ali, W.G. Dundon, G. Cattoli, C.E. Lamien);; State Central Veterinary Laboratory, Ulaanbaatar City, Mongolia (U. Ankhanbaatar, D. Purevtseren, G. Shura, B. Damdinjav)

**Keywords:** SARS-CoV-2, beaver, Mongolia, coronavirus disease, farmed animals, COVID-19, severe acute respiratory syndrome coronavirus 2, viruses, respiratory infections, zoonoses, vaccine-preventable diseases

## Abstract

We report an outbreak of COVID-19 in a beaver farm in Mongolia in 2021. Genomic characterization revealed a unique combination of mutations in the SARS-CoV-2 of the infected beavers. Based on these findings, increased surveillance of farmed beavers should be encouraged.

The COVID-19 pandemic that began in 2019 remains uncontained, and fatalities and multiple waves of infection continue to occur worldwide (*1*). The causative agent, SARS-CoV-2, has been detected in humans and several animal species, including domestic, wild, and laboratory animals (*2*,*3*). Because SARS-CoV-2 can be transmitted from humans to animals and back to humans, understanding the dynamics of infection in animals can contribute to the creation of more comprehensive response strategies.

We identified SARS-CoV-2 infection in beavers (*Castor fiber*) farmed for conservation reasons in Mongolia and report on serologic and whole genome sequence data from this outbreak. The beaver farm, located in the Bayanzurkh district in Ulaanbaatar, Mongolia, reared 32 adults and 16 kits in 2021. They were housed indoors in a large area separated by waist-high walls, with space for multiple animals. One of the 7 employees of the farm had influenza-like symptoms for several days and was diagnosed with COVID-19 on August 6, 2021. On August 9, the beaver farm reported the death of 2 beavers (one 6 months of age and one 2 years of age) after signs of coughing, nasal discharge, rasping on auscultation of the lungs and chest cavity, sluggish movement, and aversion to food. On August 13, research investigators collected nasal swabs, saliva, and 7 tissue samples (lung, kidney, liver, heart, spleen, larynx, and tongue from the 2 dead animals. Researchers also collected nasal swab specimens, saliva, and blood from 7 other beavers with notable clinical signs of coughing and purulent nasal discharge. Follow-up investigation on August 18 or 19 and on September 12 included collection of additional nasal swab specimens, saliva, and blood samples from the same animals as well as from 2 healthy animals (September 12 only).

All samples were transported to a Biosafety Level 3 facility in Ulaanbaatar and were screened by quantitative reverse transcription PCR according to the Peiris protocol (*4*). The results showed that 46 of 48 specimens from 9 animals with clinical signs, including the 2 dead animals, tested positive for SARS-CoV-2 RNA. Serum was separated from the blood samples by centrifugation (2,000 × *g* for 10 min) and stored at −20°C until required. The serum samples were then subjected to antibody screening by using a commercial ELISA kit (ID Screen SARS-CoV-2 Double Antigen Multi-species ELISA; Innovative Diagnostics, https://www.innovative-diagnostics.com). Fifteen of 23 samples tested positive and 1 was intermediate, indicating that all animals became antibody positive within 1 month of confirmation of SARS-CoV-2 RNA positivity. One clinically unremarkable beaver tested positive for SARS-CoV-2 antibodies, indicating a possible subclinical infection (Table).

**Table:** Sampling date and results of serologic analysis of SARS-CoV-2 antibodies from farmed beavers, Ulaanbaatar, Mongolia, 2021*

Animal ID	Status	Date of swab sampling and qRT-PCR results				Date of serum sampling and ELISA results†		
		2021 Aug 13	2021 Aug 19	2021 Sep 12		2021 Aug 13	2021 Aug 18	2021 Sep 12
1	Died Sep 8	Positive	NT	NT		NT	NT	NT
2	Died 2021 Sep 8	Positive	NT	NT		NT	NT	NT
3	Sick	Positive	Positive	Positive		Negative	Negative	Positive
4	Sick	Positive	Positive	Positive		Negative	Negative	Positive
5	Sick	Positive	Positive	Positive		Negative	Positive	Positive
6	Sick	Positive	Positive	Positive		Positive	Positive	Positive
7	Sick	Positive	Positive	Positive		Positive	Positive	Intermediate
8	Sick	Positive	Positive	Negative		Positive	Positive	Positive
9	Sick	Positive	Positive	Negative		Negative	Positive	Positive
10	Healthy	NT	NT	NT		NT	NT	Positive
11	Healthy	NT	NT	NT		NT	NT	Negative

*ID, identification; qRT-PCR, quantitative reverse transcription PCR; NT, not tested. †ID Screen SARS-CoV-2 Double Antigen Multi-species ELISA (Innovative Diagnostics, https://www.innovative-diagnostics.com). The interpretation is based on the signal-to-noise (S/N) ratio, (sample optical density [OD] 450/negative control OD450) × 100, according to instruction manual. Positive: S/N>100.0; intermediate: 100.0>S/N>50.0; negative: 50.0>S/N.

We shipped 5 randomly selected quantitative reverse transcription PCR–confirmed SARS-CoV-2–positive RNA samples to the Animal Production and Health Laboratory (Seibersdorf, Austria), a joint program of the International Atomic Energy Agency and the Food and Agriculture Organization of the United Nations, and subjected them to whole-genome sequencing (Appendix 1; Appendix 2). Based on genotype analysis, all 5 genome sequences were assigned to the B.1.617.2 lineage, commonly referred to as the Delta variant. At the time of sampling, Alpha and Delta variants of SARS-CoV-2 were being identified in humans in Mongolia. The closest related sequences to those we identified in the beavers studied were from human SARS-CoV-2 in Mongolia (GenBank accession nos. ON008302, OM190617, and OM961234) identified during April–September 2021 (Figure). In addition to 4 mutations in the spike region, the sequences shared 7 amino acid substitutions in open reading frame [ORF] 1a, 4 amino acid substitutions in ORF1b, and 1 amino acid substitution in nucleocapsid genes. In the beaver sequences, 4 amino acid substitutions identified were not in the human isolates from Mongolia: S2500F, A3657V in ORF1a and H604Y, T1404M in ORF1b. Although those substitutions have been identified individually in SARS-CoV-2 sequences in GenBank and the GISAID database (https://www.gisaid.org), there are no records of sequences with all 4 mutations.

**Figure:**
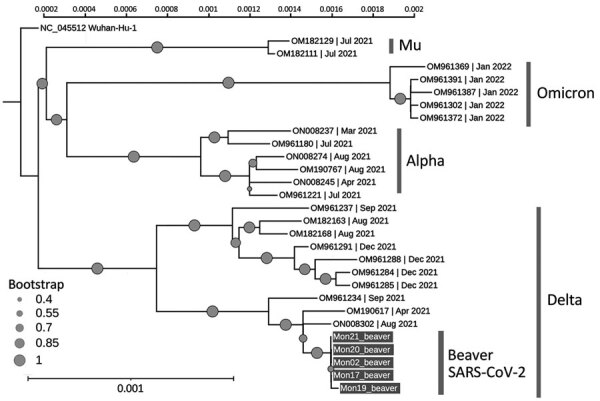
Phylogenetic tree of SARS-CoV-2 identified from beavers and humans in Mongolia (gray boxes) and reference sequences. The circle size indicates the bootstrap values at the node. The vertical bar shows the genetic distance. SARS-CoV-2 lineages are identified at right. GenBank accession numbers and date identified are shown for reference sequences; the newly obtained sequence data were deposited in GenBank (accession nos. OR389473–7).

Several cases of SARS-CoV-2 transmission between humans and animals have already been reported (*5*–*8*). An alarming aspect of SARS-CoV-2 infection in animals is that host animals can maintain the virus and contribute to the emergence in humans of new variants that have accumulated multiple mutations (*7*–*10*). Indeed, the specific combination of mutations observed in the beavers we studied has not been found in other SARS-CoV-2 sequences in public databases (as of November 2023). This finding suggests that the mutations might have occurred or accumulated after the introduction of the virus into the beaver population. Because the emergence of viruses with mutations not targeted by current SARS-CoV-2 vaccines is a credible possibility, more active surveillance of SARS-CoV-2 infection in animals should be encouraged to identify the appearance of mutated viruses. In intensively farmed animals, species–species and species–humans contact is more frequent than in animals dwelling in other environments, which might increase the risk for zoonotic pathogen transmission (*2*). Thus, implementing more active surveillance and infection control strategies is critical to disease prevention and containment.

Appendix 1 Additional information for study of SARS-CoV-2 infection in beaver farm, Mongolia, 2021.

Appendix 2 Amino acid substitutions used in study of SARS-CoV-2 infection in beaver farm, Mongolia, 2021.
