# Supplementary material for: SARS-CoV-2 Infection in Beaver Farm, Mongolia, 2021
Source: Emerg Infect Dis. 2024 Feb;30(2):391–4. doi: 10.3201/eid3002.231318 (PMC10826759; doi:10.3201/eid3002.231318)
Supplement: Appendix 1 — Additional information for study of SARS-CoV-2 infection in beaver farm, Mongolia, 2021. [file 23-1318-Techapp-s1.pdf]

Article DOI: http://doi.org/10.3201/eid3002.231318

EID cannot ensure accessibility for supplementary materials supplied by authors. Readers who have difficulty accessing supplementary content should contact the authors for assistance.

SARS-CoV-2 Infection in Beaver Farm, Mongolia, 2021

Appendix

Screening by RT-qPCR

The viral RNA was extracted using the NucleoSpin RNA Virus Mini kit (Macherey-Nagel, Germany; https://www.mn-net.com/us) from nasal swabs and saliva samples. The extracted RNA samples were screened by RT-qPCR (orf1b/nsp14 region) (*1*).

Whole genome sequencing analysis

First, RNA was reverse transcribed to cDNA using the Ion Torrent™ NGS Reverse Transcription Kit per the manufacturer's instructions. Next, the amplification of the targeted genome and the multiplexed barcoded libraries construction was performed using the Ion AmpliSeq SARS-CoV-2 Insight Research Assay GS, Chef-ready kit (Thermo Fisher Scientific, https://www.thermofisher.com/us). The kit consists of two primer pools targeting 237 amplicons of 125–275 bp, covering more than 99% of the SARS-CoV-2 genome. The combined library pool thus generated was quantified and further sequenced on a Ion S5 system (Thermo Fisher Scientific).

The run was pre-processed using the torrent suite software to remove the primers and adapters. Then, the raw reads were quality-filtered using fastq-mcf v1.04.676 (ea-utils), and their quality was assessed with FastQC (v. 011.5). De Novo Assemblies were performed using SPAdes (v3.11.1). Using the Denovo assembly's contigs, BLAST searches identified a sequence from a Mongolian human SARS-CoV-2 isolate (GenBank # ON008302) as the most relevant reference. After mapping the cleaned raw reads against the reference sequence using BWA (v0.7.17), SAMtools (v1.11) was used to generate Mpileup files, and variant calling was

performed using BCFtools (v1.9). The consensus sequences produced with vcfutils.pl (VCFtools v0.1.16) and seqtk (v1.3.106) were compared to the Denovo assemblies using Mafft (ver. 7.467) and Aliview. We obtained five consensus sequences that were subjected to genotyping, mutation detection and phylogenetic analysis. The amino acid substitutions were analyzed using Nextclade and GISAID. After manually trimming the encoding sequences in both ends, a phylogenetic tree was constructed by the Maximum-likelihood method using MEGA 7.0 software with 100 bootstrap repeats. The phylogenetic tree was visualized and annotated using iTOL.

Reference

1. World Health Organization. Detection of 2019 novel coronavirus (2019-nCoV) in suspected human cases by RT-PCR [cited 2023 Nov 1]. https://www.who.int/docs/default-source/coronaviruse/peiris-protocol-16-1-20.pdf
